# Proliferation of Perivascular Macrophages Contributes to the Development of Encephalitic Lesions in HIV-Infected Humans and in SIV-Infected Macaques

**DOI:** 10.1038/srep32900

**Published:** 2016-09-09

**Authors:** Adam R. Filipowicz, Christopher M. McGary, Gerard E. Holder, Allison A. Lindgren, Edward M. Johnson, Chie Sugimoto, Marcelo J. Kuroda, Woong-Ki Kim

**Affiliations:** 1 Department of Microbiology and Molecular Cell Biology, Eastern Virginia Medical School, Norfolk, Virginia, United States; 2 Division of Immunology, Tulane National Primate Research Center, Covington, Louisiana, United States

## Abstract

The aim of the present study was to investigate if macrophage proliferation occurs in the brain during simian immunodeficiency virus (SIV) infection of adult macaques. We examined the expression of the Ki-67 proliferation marker in the brains of uninfected and SIV-infected macaques with or without encephalitis. Double-label immunohistochemistry using antibodies against the pan-macrophage marker CD68 and Ki-67 showed that there was a significant increase in CD68+Ki-67+ cells in macaques with SIV encephalitis (SIVE) compared to uninfected and SIV-infected animals without encephalitis, a trend that was also confirmed in brain samples from patients with HIV encephalitis. Multi-label immunofluorescence for CD163 and Ki-67 confirmed that the vast majority of Ki-67+ nuclei were localized to CD163+ macrophages in perivascular cuffs and lesions. The proliferative capacity of Ki-67+ perivascular macrophages (PVM) was confirmed by their nuclear incorporation of bromodeoxyuridine. Examining SIVE lesions, using double-label immunofluorescence with antibodies against SIV-Gag-p28 and Ki-67, showed that the population of Ki-67+ cells were productively infected and expanded proportionally with lesions. Altogether, this study shows that there are subpopulations of resident PVM that express Ki-67 and are SIV-infected, suggesting a mechanism of macrophage accumulation in the brain via PVM proliferation.

Accumulation and lentiviral infection of macrophages within perivascular spaces is a fundamental concept in the pathogenesis of human immunodeficiency virus (HIV) and simian immunodeficiency virus (SIV) infection of the central nervous system (CNS). In HIV encephalitis (HIVE) and its animal model, SIV encephalitis (SIVE), the development of lesions within the brain is associated with perivascular accumulation (cuffing) of macrophages and multinucleated giant cells (MNGC)^1^,^2^,^3^,^4^,^5^. The mechanisms underlying macrophage accumulation in HIVE are not well understood. Much of the previous research aimed at elucidating mechanisms of persistent HIV infection and inflammation in the brain has focused on monocyte trafficking into the brain. Evidence supporting this, however, is lacking in both studies of HIV-infected humans and SIV-infected macaques.

It is conventionally believed that macrophages are terminally differentiated cells that are in the G0 stage of the cell cycle and do not proliferate^6^,^7^, thereby implying that macrophage accumulation in tissues is solely due to the contribution from infiltrating monocytes. However, recent mouse studies have demonstrated that macrophages do proliferate locally during inflammation^8^,^9^,^10^,^11^. These studies, using *in vivo* thymidine analog incorporation and Ki-67 co-localization, found that local macrophage proliferation dominates lesion formation and inflammation, independently of monocyte recruitment, in the pleural cavity, arterial intima, and adipose tissue.

We, therefore, sought to determine whether there are cycling cells of the macrophage lineage in the brains of adult macaques. Using double-label immunohistochemistry and multi-label immunofluorescence microscopy for various markers for macrophages (CD16, CD68, CD163, HLA-DR, or MAC387), non-macrophage lineage cells (GFAP and CNPase), cell cycle (cyclin D1, MCM2, or p16^INK4a^), cell proliferation (Ki-67 and thymidine analogs), and brain endothelial cells (GLUT1), along with SIV Gag protein (SIV p28), we present evidence that proliferating cells exist in the brains of SIV-infected macaques and that they are of a perivascular macrophage (PVM) phenotype, with the proliferation increasing along with (the degree of) encephalitis. MNGC also express these proliferation markers, with a nuclear distribution and shape such that incomplete cell division can be a mechanism other than cellular fusion for giant cell formation. We also found that the majority of these cell populations are productively infected, with an increase in the number of Ki-67+ macrophages correlating with lesion size. HIVE patient samples stained for Ki-67 and CD68 show evidence of proliferating PVM. These findings indicate that local PVM proliferation contributes to macrophage accumulation and lesion growth and may be one of the underlying mechanisms of HIV/SIV persistence in the CNS.

## Results

### Macrophage phenotype of the Ki-67+ cells in the brain and increase in Ki-67+ macrophages in macaques with SIVE

**Figure 1:**
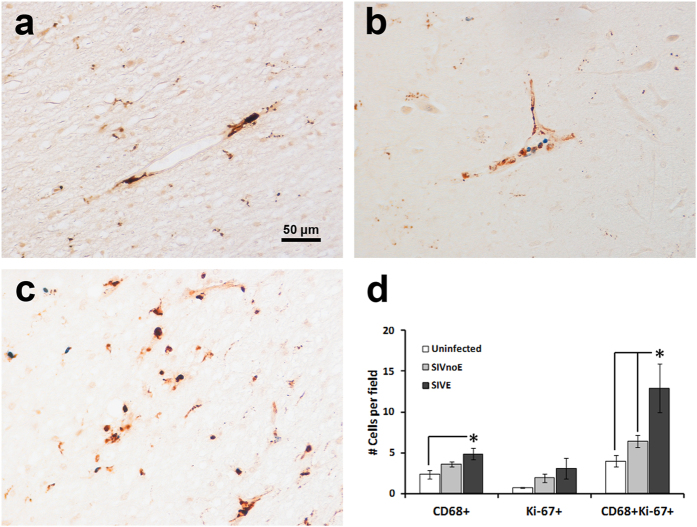
Co-expression of CD68 and Ki-67 in macrophages is associated with the vasculature and encephalitic lesions in brain tissues of rhesus macaques. Double-label immunohistochemistry for Ki-67 (blue/NBT-BCIP) and CD68 (brown/DAB) revealed the presence of Ki-67+CD68+ (blue nuclear staining and brown cytoplasm) cells around CNS vessels in uninfected (**a**), SIVnoE (**b**), and SIVE (**c**) animals. As these representative images suggest, co-expression seemed to increase going from uninfected to SIVE monkeys, and so quantification of double-positive cells was conducted (**d**). The number of CD68/Ki-67 double-positive cells was enumerated in at least 20–30 fields for each of the 9 animals examined at 100x magnification, excluding any lesions. Data expressed as mean ± SEM. The asterisk denotes significance (p < 0.05), determined by Newman-Keuls test after one-way ANOVA.

Recent studies demonstrated that local proliferation can contribute to macrophage accumulation during inflammation in the pleural cavity, arterial intima and adipose tissue^8^,^9^,^10^,^11^. We sought to investigate if there are cycling cells of the macrophage lineage in the encephalitic brains of SIV-infected adult macaques. As a first step, we examined the expression of cell cycle proteins (Ki-67, cyclin D1, and p16^INK4a^), by immunohistochemistry, in the frontal and/or temporal cortices and brainstems of uninfected control macaques (*n* = 3), SIV-infected macaques without encephalitis (SIVnoE, *n* = 4), and SIV-infected macaques with encephalitis (SIVE, *n* = 4) (Table 1). Preliminary results indicated that SIVE animals demonstrated strong nuclear immunoreactivity for these markers in the perivascular space and within encephalitic lesions compared to uninfected controls and SIVnoE animals (Fig. S1). A very similar pattern of expression of other cell cycle markers including proliferating cell nuclear antigen (PCNA) and minichromosome maintenance complex component 2 (MCM2) was observed (Fig. S2). Based on their association with inflammatory perivascular cuffs and encephalitic lesions, we suspected that cells positive for these cell cycle proteins were of the macrophage lineage. To better define this phenotype, we further explored the expression of one of these markers for cycling cells, Ki-67, along with expression of CD68, a marker useful for identifying various macrophage lineage cells. Double-label immunohistochemistry for these two markers revealed that the vast majority of cycling Ki-67+ cells were located within cells positive for CD68 in both uninfected and SIV-infected macaques (Fig. 1). These CD68/Ki-67 double-positive cells had the morphology of PVM (elongated, flattened, and found only directly adjacent to CNS vasculature) in uninfected, SIVnoE, and SIVE animals (Fig. 1a–c). Comparing uninfected and SIVnoE animals with encephalitic animals, there seemed to be an increase in double-positive cells in the encephalitic group, even when ignoring increases contributed by lesions. To quantify the difference in Ki-67+ macrophages among uninfected, infected, and encephalitic animals, we counted the number of CD68 single-positive, Ki-67 single-positive, and CD68/Ki-67 double-positive cells, enumerated in random low-power fields, while excluding any lesions, in each of the nine animals examined (Fig. 1d). The changes in CD68+ macrophages and Ki-67+ cells showed an increasing trend going from uninfected to SIVnoE to SIVE animals, but were only significant for CD68+ macrophages between uninfected and SIVE animals; however, compared to both uninfected and SIVnoE animals, the number of CD68+Ki-67+ cells in SIVE animals was significantly higher, indicating an increase in Ki-67+ macrophages in SIVE.

### Ki-67+ macrophages co-express CD163, exhibiting a PVM phenotype

**Figure 2:**
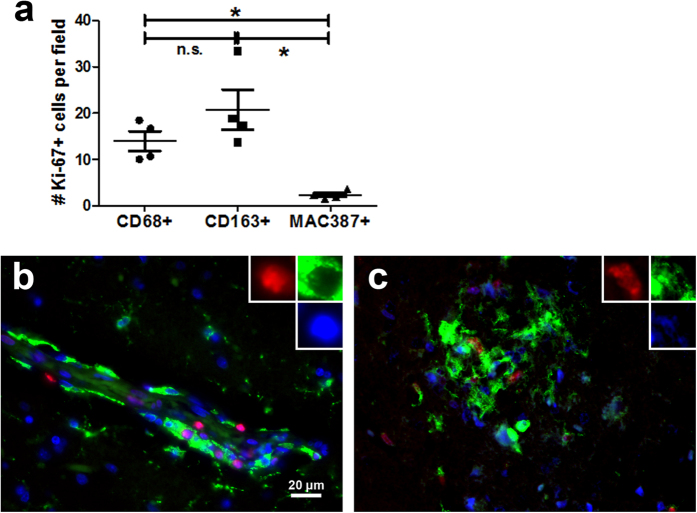
Confirmation that Ki-67 positive cells are perivascular macrophages using CD163 expression. The column scatter plot (**a**) shows quantification of Ki-67+ cells among CD68+, CD163+, or MAC387+ cell populations. In SIVE brains, there are significantly higher numbers of Ki-67+CD68+ and Ki-67+CD163+ macrophages compared to Ki-67+MAC387+ cells (*p < 0.05; two-tailed paired t-test; *n* = 4 per group). There was no significant difference between CD68+ and CD163+ macrophages in terms of co-expression of Ki-67. Counting was performed as in Fig. 1 using double-label immunohistochemical images. Triple-label immunofluorescence for Ki-67 (red), CD163 (green), and DAPI (blue) in the perivascular space (**b**) and in lesions (**c**) of SIVE macaques revealed that the majority of Ki-67+ cells also expressed CD163.

**Figure 3:**
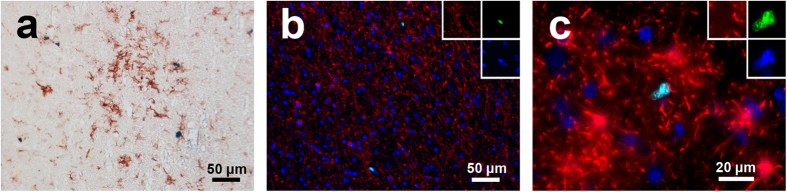
Ki-67 expression in other cell types in the SIVE brain. As in Fig. 1, double-label immunohistochemistry for Ki-67 (blue/NBT-BCIP) and HLA-DR (brown/DAB) demonstrated a minor but noticeable population of un-ramified microglia which co-expressed Ki-67 and HLA-DR (**a**). Triple-label immunofluorescence for Ki-67 (green), CNPase (**b**; red) or GFAP (**c**; red), and DAPI (blue) in SIVE macaques showed no expression of Ki-67 in oligodendrocytes (**b**) or astrocytes (**c**).

To confirm that these Ki-67+ cells were PVM and not recently infiltrated monocytes, we also examined expression of a more specific macrophage lineage marker, CD163, known to be expressed in PVM in the brain, along with MAC387, commonly used to identify infiltrated monocytes/macrophages^3^,^4^. We counted the number of CD68+Ki-67+, CD163+Ki-67+, and MAC387+Ki-67+ cells in four SIVE animals using double-label immunohistochemistry in a similar manner as for Fig. 1d. The number of both CD68+Ki-67+ and CD163+Ki-67+ PVM were significantly higher than that of MAC387+Ki-67+ cells in these encephalitic animals (Fig. 2a). Multi-label immunofluorescence for CD163 and Ki-67 with a DAPI nuclear counterstain indeed revealed that the majority of Ki-67+ cells were CD163+ macrophages found both in the perivascular cuffs (Fig. 2b) and encephalitic lesions (Fig. 2c) of SIV-infected macaques. The minor subset of CD68−CD163−MAC387+Ki-67+ were within perivascular spaces and lesions with a morphology similar to infiltrated monocytes, previously described elsewhere^4^ (Fig. S3a,c). Interestingly, scattered MAC387+Ki-67+ cells were also found in both the brain parenchyma and the lumen of vessels. These cells displayed the morphology of polymorphonuclear leukocytes and represented the majority of MAC387+Ki-67+ cells found (Fig. S3b). These cells were almost exclusively Ki-67+ and may represent a population of infiltrating granulocytes. We additionally examined other brain cell types to determine if there were any other minor subsets of Ki-67+ cells (Table 2; Fig. 3). Double-label immunohistochemistry or immunofluorescence for HLA-DR, GFAP, or CNPase and Ki-67 revealed that activated microglia (Fig. 3a) represented a small subset of Ki-67+ cells found in the parenchyma, but that oligodendrocytes and astrocytes did not (Fig. 3b,c). Altogether, while the vast majority of Ki-67+ cells are CD68+CD163+ PVM, there are minor populations of other cell types, most noticeably recently infiltrating monocytes, polymorphonuclear cells, and activated microglia, which are also positive for Ki-67.

### *In vivo* incorporation of thymidine analogs confirms the proliferative state of Ki-67+ macrophages

Having shown that a population of Ki-67+ PVM exists in the brains of adult macaques, we sought to confirm that this was an actively proliferating population. Since Ki-67 is present during all active phases of the cell cycle^12^, expression of Ki-67 does not necessarily indicate that a cell is undergoing cell division, but rather that it has the ability to proliferate. Indeed, when DNA synthesis is blocked, cells remain positive for Ki-67 even though the cell division cycle has been arrested, as measured by BrdU incorporation^13^. Therefore, we used multi-label immunofluorescence on tissues from animals which had received BrdU and EdU injections to test whether Ki-67+ cells were co-labeled with these thymidine analogs. Typically, SIV-infected macaques had received one single i.v. injection of BrdU early between day 5 and day 10 post infection and the other single injection of EdU 2 days prior to euthanasia, which is between 55 and 300 days post infection (see Table S1 for experimental injection timeline).

**Figure 4:**
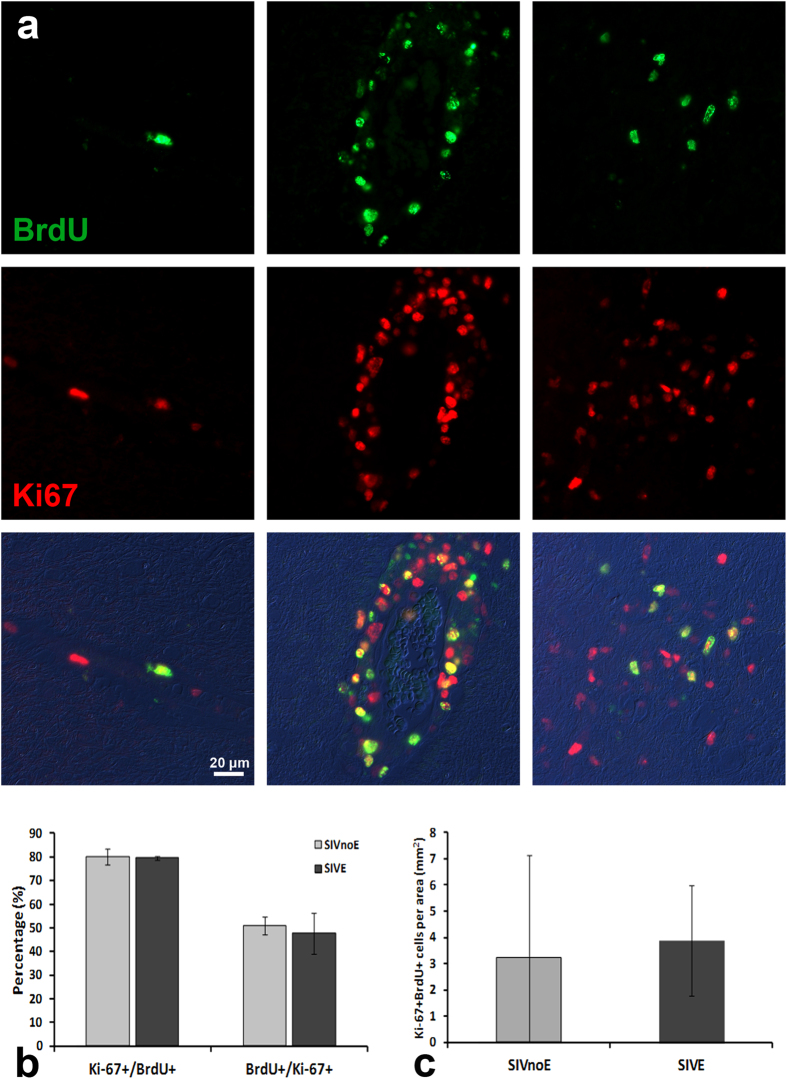
Verification that Ki-67 positivity represents proliferation using incorporation of the thymidine analog BrdU. Double-label immunofluorescence (**a**) for Ki-67 (red) and BrdU (green) in SIVnoE (left column) and SIVE (middle and right columns) macaques revealed co-localization in the nuclei of cells in the perivascular space (left and middle columns) and encephalitic lesions (right column). The arrowheads in the middle and right columns indicate double-labeled mitotic figures. Ki-67-postive and BrdU-labeled cells in the cortex were counted for both the SIVnoE and SIVE groups (ranging from 216 to 3,436 cells per animal, *n* = 3 per group). The mean percentage of BrdU-labeled cells that were positive for Ki-67 (Ki-67+/BrdU+) and the mean percentage of Ki-67+ cells that were labeled with BrdU (BrdU+/Ki-67+) are plotted for both SIVnoE and SIVE groups (**b**). The mean numbers of double-positive cells are plotted for SIVnoE and SIVE groups (**c**). Error bars denote standard deviations (**b,c**). No significance between SIVnoE and SIVE (p > 0.05).

When we examined BrdU incorporation, we found evidence of BrdU+Ki-67+ double-labeled nuclei restricted to areas surrounding vasculature in both SIVnoE and SIVE animals (Fig. 4). Occasionally, we observed double-labeled mitotic figures associated with CNS vessels and within the lesions of SIVE animals. In all tissues examined, the amount of both Ki-67 expression and co-labeling of BrdU appeared to increase in SIVE compared to SIVnoE animals, consistent with our previous findings (Fig. 4a, left versus middle columns). Quantitative analysis of the BrdU+ cells in the cortical areas of these animals revealed that, while not all of the Ki-67+ cells were labeled with BrdU, the vast majority of BrdU+ cells were stained for Ki-67 (Fig. 4b). This followed our expectations, as BrdU was administered by a 30-min infusion and is specific to the S phase, while Ki-67 is expressed in all but the resting phase. Co-labeling was also observed in lesions of SIVE animals (Fig. 4a, right column). Animals which had received EdU injections displayed a similar pattern of expression (Fig. S4). Incorporation of these two thymidine analogs in cells which co-express Ki-67 suggests that not only does a population of PVM and meningeal macrophages with a proliferative potential exist, but that at least a portion of these cells are actively dividing, or at least undergoing chromosomal replication in preparation for cellular division. Additionally, we found a rare subpopulation of BrdU/EdU double-labeled cells around the vasculature in animals that had received injections of BrdU and EdU at different time points (Table S1; Fig. S5), indicating that some of these proliferating cells may be long-lasting and capable of self-renewal.

### SIV-infected MNGC express proliferation markers

**Figure 5:**
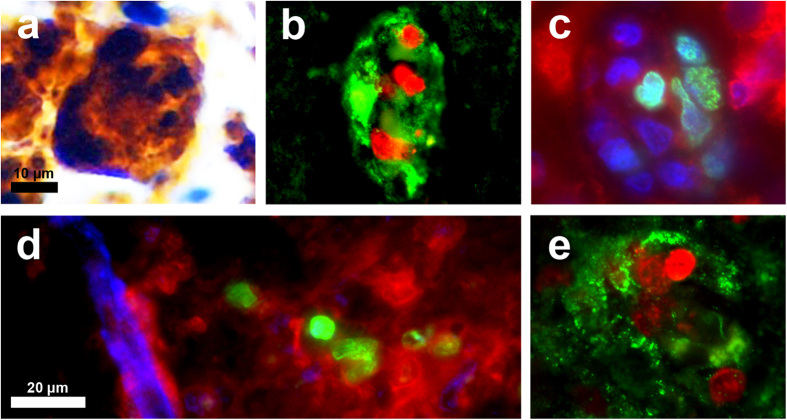
MNGC show a proliferative capacity and are productively infected. Double-label immunohistochemistry for Ki-67 (blue/NBT-BCIP) and CD68 (brown/DAB) revealed the presence of Ki-67+ nuclei within an MNGC (**a**). Double-label immunofluorescence for Ki-67 (red) and CD163 (green) confirmed that these were indeed MNGC likely originating from PVM (**b**). Triple-label immunofluorescence for CD16 (red), BrdU (green) and either DAPI (**c**; blue) or Glut-1 (**d**; blue) elucidated the fact that some, though not all, nuclei within an MNGC were actively going through DNA synthesis both within lesions and near vessels. Double-label immunofluorescence for Ki-67 (red) and SIVp28 (green) revealed that MNGC, known to harbor virus, are able to have a proliferative capacity (**e**).

Besides perivascular accumulation of macrophages, one of the defining features of HIVE/SIVE is the formation of MNGC. We were curious, then, whether these cells, traditionally held to form from the fusion of macrophages, also expressed markers of proliferation. Upon close examination, we noted that almost all nuclei inside CD68+ MNGC located perivascularly and within encephalitic lesions were positive for Ki-67 (Fig. 5a). This same expression pattern was found in MNGC stained for CD163 (Fig. 5b). When examined for BrdU incorporation, MNGC within lesions (Fig. 5c) and around vessels (Fig. 5d) contained some nuclei labeled with BrdU, as expected. In Fig. 5c, a MNGC expressing CD16 (Fc gamma receptor III) was packed with DAPI-positive nuclei, some of which were co-labeled with BrdU. In addition, mitotic figures in MNGC were infrequently found. Interestingly, the distribution of BrdU-positive nuclei within these MNGC was not around the perimeter of the cell as would be expected if they formed from the fusion of distinct macrophages; instead, almost all of the MNGC showed proliferating nuclei within the center, sometimes spreading to the perimeter. Altogether, this strongly suggests that MNGC of encephalitic brains may form from nuclear division without cell division rather than by cell-to-cell fusion. MNGC are known to harbor significant amounts of HIV/SIV^14^, and so expression of the structural protein SIV Gag p28 was examined within these cells, revealing that the majority were both productively infected and proliferating (Fig. 5e).

### PVM proliferation is correlated with increased size of encephalitic lesions

**Figure 6:**
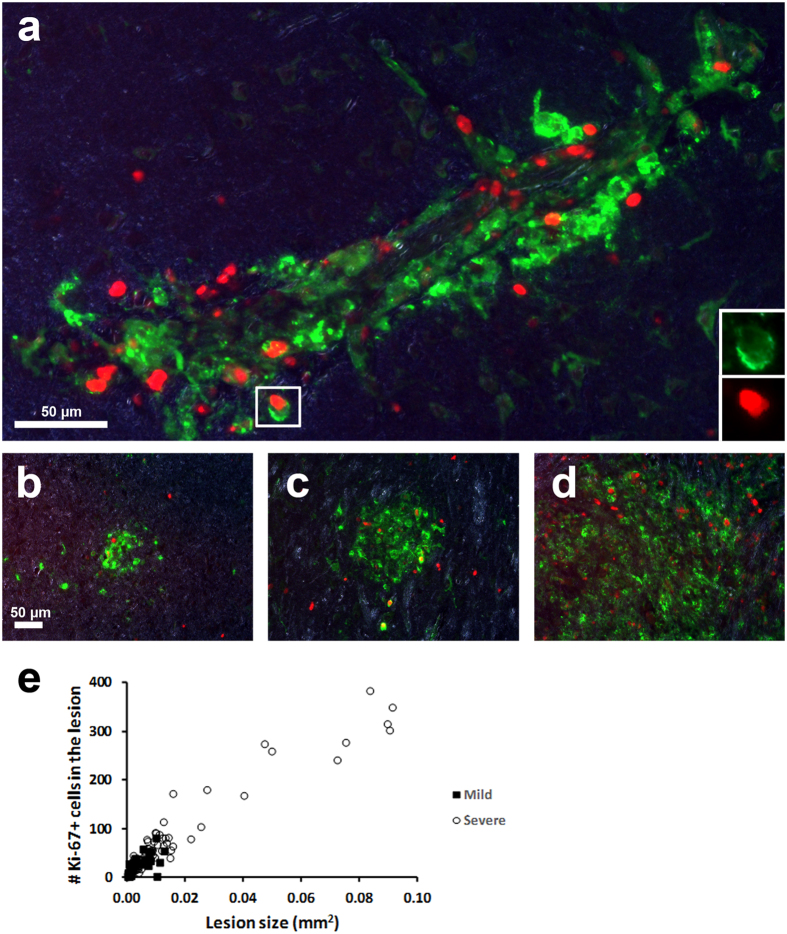
Proliferating PVM are productively infected around vessels and within encephalitic lesions. Double-label immunofluorescence for Ki-67 (red) and SIVp28 (green) in SIVE macaques in both the perivascular space (**a**) and lesions (**b–d**) confirmed that PVM in general have the capacity to proliferate. A further observation was made that the number of Ki-67-expressing cells seemed to increase as lesions grew in size. Quantification of this relationship revealed a significant correlation between lesion size and the number of Ki-67+ cells within a lesion (r = 0.9292, p < 0.0001) (**e**). Furthermore, cases of severe SIVE (open circles) tended to produce larger lesions, with a corresponding increase in Ki-67 expression, compared to milder cases (closed squares).

With evidence of productively infected, proliferating MNGC in hand, we next tackled the question of whether or not proliferating PVM in general are productively infected. As has been shown before, macrophages are a major reservoir for latent and productive HIV/SIV infection in the CNS^15^,^16^,^17^; additionally, encephalitic lesions consist predominantly of macrophages^3^. We thus speculated that the population of proliferating macrophages may contribute to SIVE lesion formation. Multi-label immunofluorescence for Ki-67 and SIVp28 revealed that a majority of Ki-67+ cells were indeed infected (Fig. 6). In particular, we found evidence of productively infected, proliferating macrophages both around vessels (Fig. 6a) and within lesions (Fig. 6b–d). Not all of the Ki-67+ cells were positive for SIV, nor were all of the SIV p28+ macrophages Ki-67-positive, although we noted that as lesions grew in size, there seemed to be a corresponding increase in Ki-67+SIV-p28+ cells. Quantifying this relationship, we indeed found that the number of Ki-67+ cells within a lesion significantly correlated with lesion size (Fig. 6e; *n* = 127, *r* = 0.9292, *p* < 0.0001, Spearman correlation). This suggests that proliferation of macrophages plays a significant role in the formation of encephalitic lesions. In support of this notion, when comparing mild and severe cases of SIVE, we noted a large increase in Ki-67+ cells and thus, as expected, lesion sizes in severe SIVE tissues. Taken together, these results suggest proliferating macrophages may serve as a reservoir for SIV in the CNS, accounting for the persistence of the virus in the brain, leading to encephalitis.

### CD68+ macrophages display increased expression of Ki-67 in HIVE subjects

**Figure 7:**
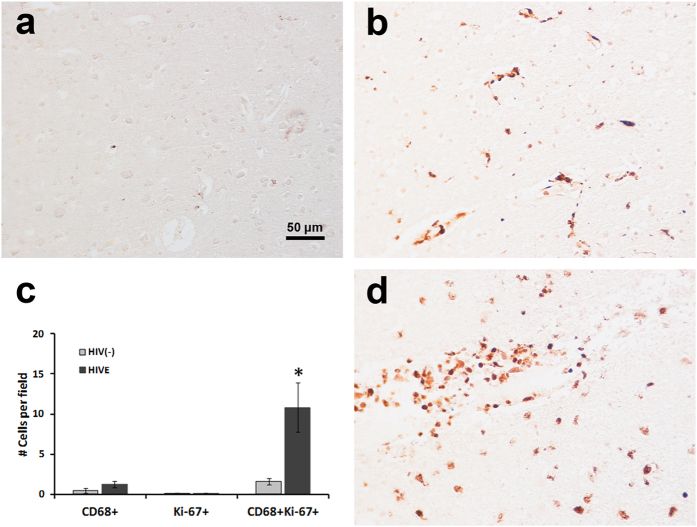
Evidence of PVM proliferation in human brains. As in Fig. 1, double-label immunohistochemistry confirmed the presence of Ki-67+ CD68+ macrophages (blue nuclear staining and brown cytoplasm) in the adult human brain (**a,b,d**). The number of Ki-67+ brain PVM in HIVE patients (**b**) was significantly higher than in HIV seronegative controls (**a**) (*p < 0.05; two-tailed, unpaired t-test; *n* = 3 per group; (**c**). Data expressed as mean ± SEM. In a human subject with CAA the number of Ki-67+ macrophages also demonstrated a similar increase (**d**).

To better solidify our findings in the monkey model, we investigated whether Ki-67+ cells were also positive for CD68 in subjects diagnosed with HIVE, the pathological correlate of HIV dementia (Table 3). Cortical tissues from HIV seronegative subjects (*n* = 4) and HIV-infected subjects with HIVE (*n* = 3) were stained for Ki-67 and CD68 using double-label immunohistochemistry (Fig. 7). Staining revealed a similar occurrence to the monkey model, previously stated, where the majority of Ki-67+ cells were also positive for CD68 in HIVE brain samples. These cells appeared to be flat, elongated, and the majority of them were found adjacent to CNS vasculature, which is characteristic of the PVM. Quantitative analysis was performed by counting the number of CD68 single-positive, Ki-67 single-positive, and CD68/Ki-67 double-positive cells, enumerated in 20 random, low-power fields per subject, avoiding lesions (Fig. 7c). When comparing CD68 single-positive and Ki-67-single positive, there appeared to be little difference between the HIVE (Fig. 7b) and uninfected (Fig. 7a) subjects. However, when comparing the number of CD68/Ki-67 double-positive cells, the HIVE subjects had significantly higher numbers of Ki-67+ macrophages than the uninfected subjects. Interestingly, in a subject diagnosed with cerebral amyloid angiitis (CAA) we found a similar increase in Ki-67+ macrophages (Fig. 7d). In summary, these findings confirm that there are cycling cells of the macrophage lineage in the human brain, as suggested by our SIV/macaque model, which may contribute to various brain pathologies including HIVE and CAA.

## Discussion

Determining the exact mechanisms behind perivascular accumulation of macrophages leading to a potential reservoir for persistent infection would be a major step in understanding AIDS neuropathogenesis. Infection of the CNS by HIV remains one of the most pressing problems in HIV research and treatment. Both human and macaque models support the notion of the brain as a reservoir for persistent infection^18^,^19^. The existence of a CNS viral reservoir presents a twofold problem: viral persistence even with highly active antiretroviral therapy (HAART) leading to viral rebound once treatment is stopped, as well as continued cognitive decline related to HIV infection. Two distinct populations of brain macrophages are thought to comprise the majority of this viral reservoir: PVM and parenchymal microglia, both of which have been shown to play key roles in the neurologic manifestations of AIDS^15^,^16^,^20^,^21^. In particular, PVM are the major cell type productively infected with HIV and its nonhuman counterpart, SIV, in the brain^15^,^16^,^20^,^22^,^23^,^24^,^25^,^26^. HIVE, the pathological correlate of HIV-associated dementia (HAD), is characterized by a perivascular accumulation of macrophages and MNGC in the brain. The earliest uses of antiretroviral therapy showed that the symptoms of HIV encephalopathy could be reversed in some patients with treatment that reduced the plasma viral load^27^. However, even with HAART, while the incidence of HAD has decreased, the prevalence of milder forms of cognitive impairment has increased^28^. To go along with this, even after HAART, levels of microglial/macrophage activation remain comparable with levels seen, pre-HAART, in HIVE and AIDS cases^29^.

It is commonly held that bone marrow-derived blood monocytes continuously traffic to the CNS and become PVM under normal conditions, with acceleration of traffic taking place during inflammation and infection, which leads to immune activation of monocytes/macrophages^30^,^31^. The importance of monocyte trafficking is highlighted by the fact that, in the SIV macaque model of AIDS, use of BrdU, an analog of thymidine that is incorporated in DNA during replication, revealed that increased monocyte turnover correlates with the severity of SIVE^32^. Additionally, using genetically modified CD34+ hematopoietic stem cells, Williams and colleagues showed that a population of brain PVM are slowly renewed by CD34+ hematopoietic stem cell-derived precursors in rhesus macaques that received total-body gamma-irradiation before transplantation^33^. Conversely, past studies examining PCNA expression in SIV and HIV encephalitis found evidence of infected perivascular macrophages that were PCNA-positive^2^,^34^. As its name suggests, PCNA is often used as a marker for cellular proliferation; however, it is also expressed in non-dividing cells undergoing DNA synthesis and repair^35^,^36^,^37^. In these studies, a lack of Ki-67/MIB-1 expression and BrdU labeling, then, were used to suggest that alterations in monocyte/macrophage trafficking and turnover but not macrophage proliferation play the most significant role in the pathogenesis of SIVE/HIVE.

Despite these findings, and encouraged by recent evidence of proliferating macrophages in other pathophysiological settings, we set out to determine whether proliferation plays any role in HIV/SIV infection of the brain. By employing a new rabbit monoclonal antibody against Ki-67, clone SP6, which has been shown to have an enhanced sensitivity and intensity when compared to mouse monoclonal antibody MIB-1^38^,^39^,^40^, and which we ourselves found to have better immunoreactivity in macaque tissue samples (Fig. S6), we were able to show the existence of Ki-67+ macrophages in the brain. Using this as a launching point, we conducted further immunohistochemical imaging of these cells using macrophage, proliferation, and SIV markers to investigate if macrophage proliferation is a mechanism for macrophage accumulation and viral persistence in SIVE. Quantification of the CD68+Ki-67+ macrophages revealed a significant increase in the population size of these cells in SIVE animals. The majority of these cells were confirmed, using fluorescent microscopy, to be PVM via their co-expression of CD163 and by their location around Glut1+ CNS vessels. Further phenotyping with MAC387 and numerical comparison with the CD68+ and CD163+ macrophage populations confirmed that PVM represented the majority of the Ki-67-positive population, with the minority being CNS-infiltrating monocytes and polymorphonuclear leukocytes. *In vivo* labeling assays with BrdU and EdU confirmed that Ki-67 expression represented a true proliferative population. This population includes MNGC, a specific feature of encephalitis. Importantly, this proliferating population of PVM and MNGC were shown to be productively infected, expanding along with the degree of encephalitis and as lesions grew in size. Finally, evidence of this Ki-67+ PVM population was found in HIVE patient samples. HIV/SIV infection per se does not seem to directly drive macrophage proliferation since proliferating brain macrophages were also found in the brain of a CAA patient who was HIV-negative. It was previously shown that IL-4, favoring M2 macrophage polarization, directly activates tissue-resident macrophages to proliferate *in vivo*^8^,^41^. Very interestingly, IL-4 was also previously shown as a biomarker for simian-human immunodeficiency virus encephalitis^42^,^43^,^44^,^45^. It is thus possible that IL-4-driven neuroinflammation is responsible for proliferation of perivascular macrophages.

Previous studies have hinted that recruitment of monocytes from the periphery might not be the dominant mechanism underlying encephalitic lesion formation. For example, Soulas *et al*. found that few MAC387+ cells, representing recently infiltrated monocytes, were in the CNS of acutely infected macaques (day 21 post infection) while they were present in SIVE animals, thereby indicating that infiltration of MAC387+ monocytes into the CNS occurs only late in disease progression^4^. Furthermore, characterizing the cellular makeup and macrophage phenotype of encephalitic lesions, the authors revealed that in animals with mild encephalitis the percentage of MAC387+ cells is increased, whereas in severe encephalitis CD68+ cells, representing resident macrophages, were instead relatively more numerous. As another example, Clay *et al*. found that when examining the migration of adoptively transferred dye-labeled monocytes, acutely SIV-infected macaques averaged only about one labeled CD16+ monocyte per section examined^46^. Even more direct evidence comes from a recent study which clearly demonstrated that around 82% of CD163+ macrophages found later in SIVE lesions were already present in the CNS at day 20 post infection (the time of the first intracisternal injection of dextran dyes to selectively label pre-existing perivascular macrophages), a time point when no significant monocyte infiltration was found, and well before the development of encephalitis^5^. These data, along with the results presented here, support a notion of early seeding of resident PVM followed by proliferation, rather than recruitment of monocytes from the periphery as the primary mechanism of late encephalitic lesion formation and persistence of the viral reservoir in the brain.

While future studies, including *in vitro* proliferation assays and further immunohistology of HIVE tissue samples will be necessary, our data presented here serves as a first step towards illustrating that macrophage proliferation may be critically important to a better understanding of the mechanisms responsible for macrophage accumulation and the formation of encephalitic lesions.

## Methods

### Animals

A total of 13 adult, male rhesus macaques (*Macaca mulatta*) were used in the study. All animals were housed at the Tulane National Primate Research Center (TNPRC). All procedures of this study were approved by the Tulane University Institutional Animal Care and Use Committee, and were carried out in accordance with the National Institutes of Health “Guide for the Care and Use of Laboratory Animals” and the recommendations of the Weatherall report, “The use of non-human primates in research”. All possible measures were taken to minimize discomfort of the animals. For all routine procedures such as blood collection and physical examination, animals were fully anesthetized with ketamine HCl under the direction of a veterinarian. Animals were humanely euthanized by the veterinary staff at the TNPRC in accordance with its endpoint policy. The criteria for euthanasia include 15% weight loss in two weeks, unresponsive opportunistic infection, persistent anorexia, severe intractable diarrhea, progressive neurological signs, significant cardiac and/or pulmonary signs, or any other serious illness. Euthanasia was conducted by anesthesia with ketamine HCl (10 mg/kg) followed by an overdose with sodium pentobarbital. This method is consistent with the recommendation of the American Veterinary Medical Association Guidelines on Euthanasia. Archived tissues from 3 uninfected rhesus macaques were included as normal, uninfected controls. The animals used in the present study are listed in Table 1.

### Macaque tissue samples

Formalin-fixed, paraffin-embedded necropsy brain tissues sections (5 μm thick) of the frontal, parietal, and temporal cortices, as well as brainstem, from adult, male rhesus macaques (*Macaca mulatta*) were used. Several of these archived specimens were from previous studies involving *in vivo* labeling with thymidine analogs. In total, tissues from thirteen monkeys, three uninfected control monkeys and nine monkeys intravenously infected with SIVmac251 virus (20 ng of SIV p27), were used. They were not perfused at necropsy. Evidence of SIVE in six of the infected monkeys was defined by the presence of SIV proteins in the brain and the accumulation of macrophages and MNGC.

### Human tissue samples

Formalin-fixed, paraffin-embedded sections of parietal, temporal and occipital cortices were obtained from the Manhattan HIV Brain Bank (MHBB), a member of the National NeuroAIDS Tissue Consortium. A total of 3 adult HIVE cases, and 4 seronegative controls were examined (Table 3).

### *In vivo* BrdU/EdU labeling and detection

The thymidine analogs 5-bromo-2′-deoxyuridine (BrdU; Sigma-Aldrich, St. Louis, MO) and 5-ethynyl-2′ deoxyuridine (EdU; Molecular Probes, Eugene, OR), were used to pulse-label cells undergoing DNA synthesis as previously described^47^. Supplementary Table 1 describes the infusion schedule for each animal. Dual-pulse labeling in brain tissues was assessed by immunofluorescence using an anti-BrdU monoclonal antibody that did not cross-react with EdU. EdU was detected using the Click-iT EdU Alexa Fluor 488 Imaging Kit (see below for details).

### Immunohistochemistry

Immunohistochemistry was performed using the antibodies listed in Table 2, as previously described^47^. After incubation for 1 h at 58–60 °C, sections were deparaffinized and rehydrated. Sections were then pretreated for antigen retrieval with a citrate-based Antigen Unmasking Solution (Vector Laboratories, Burlingame, CA) in a microwave (900 W) for 20 min. After cooling for 20 min, sections were washed with Tris-buffered saline (TBS) containing 0.05% Tween-20 for 5 min, followed by incubation with a peroxidase blocking solution (Bloxall, Vector Laboratories) for 5 min. After washing again, sections were incubated with either 5% normal horse or goat serum in TBS for 15 and 30 min, respectively, followed immediately by primary antibody incubation for 1 h at room temperature or overnight at 4 °C. After washing, sections were incubated with a biotinylated secondary antibody (Vector Laboratories) for 30 min. Dako Antibody Diluent (Dako, Carpinteria, CA) was used for both primary and secondary antibody dilution. Following another wash, sections were incubated for 30 min with an avidin-biotin peroxidase complex (Vectastain ABC Elite kit, Vector Laboratories) and developed with diaminobenzidine (DAB; Dako) with Mayer’s Hematoxlyin (Dako) used as a nuclear counterstain. Sections were dehydrated and mounted using VectaMount (Vector Laboratories). To examine the phenotype of Ki-67+ brain cells, double-label immunohistochemistry for CD68, CD163, HLA-DR, MAC387, GFAP or CNPase with Ki-67 was performed using both Vectastain Elite ABC and ABC-alkaline phosphatase kits, according to the manufacturer’s instructions. For these sections, Ki-67 was developed using NBT/BCIP (Roche, Basel, Switzerland) with DAB being used for the cell type-specific markers. Sections were visualized using a Nikon Coolscope digital microscope and, for CD68, CD163, or MAC387 and Ki-67 counting, analyzed using a computer-assisted image processing and analysis software (ImageJ, NIH).

### Immunofluorescence microscopy

Double- or triple-label immunofluorescence was done to further phenotype proliferating cells in the brain, to examine co-labeling with thymidine analogs, and to determine whether proliferating cells were productively infected. As described above, sections were deparaffinized and rehydrated, followed by antigen retrieval. After washing with phosphate-buffered saline (PBS) containing 0.2% fish skin gelatin (FSG; Sigma-Aldrich), sections were permeabilized with PBS containing 0.2% FSG and 0.1% Triton X-100 for 1 h. Following another wash, sections were incubated with 5% normal goat serum in PBS for 30 min at room temperature before incubation for 1 h at room temperature or overnight at 4 °C with primary antibodies diluted in PBS/FSG. After primary antibody incubation, the sections were washed in PBS/FSG and incubated with an Alexa Fluor 350-, 488-, 555-, or 594- conjugated secondary antibody (Molecular Probes; diluted at 1:1000 in PBS/FSG) for 1 h at room temperature. The sections were washed with PBS/FSG before the addition of the next primary antibody. Animals which had received intravenous (i.v.) infusions of BrdU (60 mg/kg) and/or EdU (50 mg/kg) were also assessed for labeling by immunofluorescence. Detection of EdU was done using a Click-iT EdU Alexa Fluor 488 kit (Molecular Probes) according to the manufacturer’s instructions. After immunofluorescence staining, the sections were treated with 10 mM CuSO_4_ in 50 mM ammonium acetate buffer for 45 min to quench autofluorescence. The sections were rinsed in distilled water, and cover slipped with Aqua-Mount aqueous mounting medium (Thermo Scientific, Waltham, MA). A Zeiss Axio Observer.Z1 fluorescence microscope was used to analyze the fluorescent labeled sections. Zeiss AxioVision Release 4.8.2 was used to capture and merge fluorescence images. Adobe Photoshop CS12.1 was also used to merge layers into a single image. ImageJ was used for quantification of BrdU/Ki-67 and Ki-67/SIVp28 double-labeling. For the latter, SIVE lesions were measured and the Ki-67+ cells were counted within each lesion. A total of 127 lesions were included in the analysis.

### Statistical analysis

Newman-Keuls test in conjunction with one-way ANOVA or two-tailed, unpaired t-test with Welch’s correction were used to determine significance in the quantification of CD68+Ki-67+ cells (enumerated in at least twenty to thirty random fields of each tissue used at a magnification of 100X) in SIVE and HIVE, respectively. Two-tailed, paired t-test was used to compare Ki-67 expression in different myeloid populations. The Spearman correlation coefficient was calculated to determine the relationship between lesion size and the number of Ki-67+ cells in the lesion. A two-tailed *p*-value was presented.

## Additional Information

**How to cite this article**: Filipowicz, A. R. *et al*. Proliferation of Perivascular Macrophages Contributes to the Development of Encephalitic Lesions in HIV-Infected Humans and in SIV-Infected Macaques. *Sci. Rep.* **6**, 32900; doi: 10.1038/srep32900 (2016).

## Supplementary Material

Supplementary Information

## Figures and Tables

**Table 1:** Animals Used in the Present Study.

Monkey group (size)	Animal number	Age at euthanasia (years)	Survival after infection (days)	Degree of SIV-induced encephalitis
Uninfected controls (n = 3)				
	EC61	8.99	n/a	n/a
	GI53	5.02	n/a	n/a
	GI84	5.41	n/a	n/a
SIV-infected without encephalitis (n = 4)				
	DR28	8.84	114	n/a
	DR67	9.16	49	n/a
	GL96	3.35	49	n/a
	EM89	8.19	70	n/a
SIV-infected with encephalitis (n = 6)				
	CN66	20.88	68	Mild
	CV39	10.68	147	Severe
	DG09	10.28	98	Mild
	FG54	5.70	168	Moderate/severe
	GN24	5.15	69	Mild
	HD42	5.62	91	Severe

n/a, not applicable.

**Table 2:** Antibodies Used in the Present Study.

Antigen	Clone	Isotype	Reactivity	Manufacturer	Application
BrdU	BU1/75	Rat IgG2a	n/a	Serotec	IF
BrdU	Bu20a	Mouse IgG1	n/a	BioLegend	IF
BrdU	MoBu-1	Mouse IgG1	n/a	BioLegend	IF, FC
BrdU	ZBU30	Mouse IgG1	n/a	Invitrogen	IF
CD16	2H7	Mouse IgG2a	Hu, Mk	Novocastra	IF
CD68	KP1	Mouse IgG1	Hu, Mk	NeoMarkers	IHC, IF
CD163	10D6	Mouse IgG1	Hu, Mk	NeoMarkers	IHC, IF
CD163	EDHu-1	Mouse IgG1	Hu, Mk	Serotec	IF
CNPase	11-5B	Mouse IgG1	Hu, Mk, Rb, Rt, Ms	Millipore	IF
Cyclin D1	SP4	Rabbit IgG	Hu, Mk	NeoMarkers	IHC
GFAP	5C10	Mouse IgG1	Hu, Mk, Rt, Ms	BioLegend	IF
Glut1	Polyclonal	Rabbit IgG	Hu, Mk, Rt	NeoMarkers	IF
HLA-DR+DP+DQ	CR3/43	Mouse IgG1	Hu, Mk	Invitrogen	IHC
Ki-67	MIB-1	Mouse IgG1	Hu, Mk	Santa Cruz	IHC
Ki-67	SP6	Rabbit IgG	Hu, Mk	Vector Laboratories	IHC, IF
MCM2	D7G11	Rabbit IgG	Hu, Mk	Cell Signaling	IHC
MRP8/MRP14	MAC 387	Mouse IgG1	Hu, Mk	NeoMarkers	IF
p16	JC8	Mouse IgG2a	Hu, Mk	Santa Cruz	IHC
PCNA	PC10	Mouse IgG2a	Rt, Ms, Hu, Mk	Santa Criz	IHC
SIV p28	3F7	Mouse IgG1	n/a	Fitzgerald	IF

n/a, not applicable; Hu, human; Mk, monkey; Rb, rabbit; Rt, rat; IHC, immunohistochemistry; IF, immunofluorescence; FC, flow cytometry.

**Table 3:** Human Subjects.

PID	HIV-1 status	CNS pathology	Age/gender	Risk factor
MHBB 76	+	HIVE	38 F	IVDU
MHBB 87	+	HIVE	31 M	Heterosexual
MHBB 537	+	HIVE	45 F	IVDU
MHBB 101	−	None	59 F	None known
MHBB 104	−	None	56 F	None known
MHBB 109	−	None	56 M	None known
A06-28	−	Cerebral amyloid angiitis	57 F	None known

IVDU, intravenous drug user.
