# Supplementary material for: Proliferation of Perivascular Macrophages Contributes to the Development of Encephalitic Lesions in HIV-Infected Humans and in SIV-Infected Macaques
Source: Sci Rep. 2016 Sep 9;6:32900. doi: 10.1038/srep32900 (PMC5017189; doi:10.1038/srep32900)
Supplement: Supplementary Information [file srep32900-s1.pdf]

SUPPLEMENTARY MATERIAL

Scientific Reports

Proliferation of Perivascular Macrophages Contributes to the Development of Encephalitic Lesions in HIV-Infected Humans and in SIV-Infected Macaques

Adam R. Filipowicz[1], Christopher M. McGary[1], Gerard E. Holder[1], Allison A. Lindgren[1], Edward M. Johnson[1], Chie Sugimoto[2], Marcelo J. Kuroda[2], Woong-Ki Kim[1,*]

[1]*Department of Microbiology and Molecular Cell Biology, Eastern Virginia Medical School, Norfolk, Virginia, United States;* [2]*Division of Immunology, Tulane National Primate Research Center, Covington, Louisiana, United States*

[*]Corresponding author at: Department of Microbiology and Molecular Cell Biology, Eastern Virginia Medical School, 700 W. Olney Road, Lewis Hall 3174, Norfolk, VA 23501, USA. Tel.: 757-446-5639. *E-mail address:* kimw@evms.edu (W.-K. Kim)

Table S1 Animals Used for In Vivo BrdU/EdU Labeling

Animal	DNA label injection schedule (days prior to sacrifice)	Duration of infection (days)	CNS pathology
CV39	BrdU (-142), EdU (-51), BrdU (-2)	147	Severe SIVE
DG09	BrdU (-90), EdU (-51), BrdU (-2)	98	Mild SIVE
DR28	BrdU (-109), EdU (-18), BrdU (-2)	114	SIVnoE
DR67	BrdU (-41), EdU (-2)	49	SIVnoE
EC61	EdU (-7), BrdU (-3)	n/a	Normal uninfected
EM89	BrdU (-62), EdU (-20)	70	SIVnoE
GI53	EdU (-7), BrdU (-3)	n/a	Normal uninfected
GI84	BrdU (-2)	n/a	Normal uninfected
GL96	BrdU (-2)	49	SIVnoE
GN24	BrdU (-61), EdU (-19)	69	Mild SIVE

[a]SIVE, SIV encephalitis
[b]SIVnoE, no sign of encephalitis

Supplementary Fig. 1

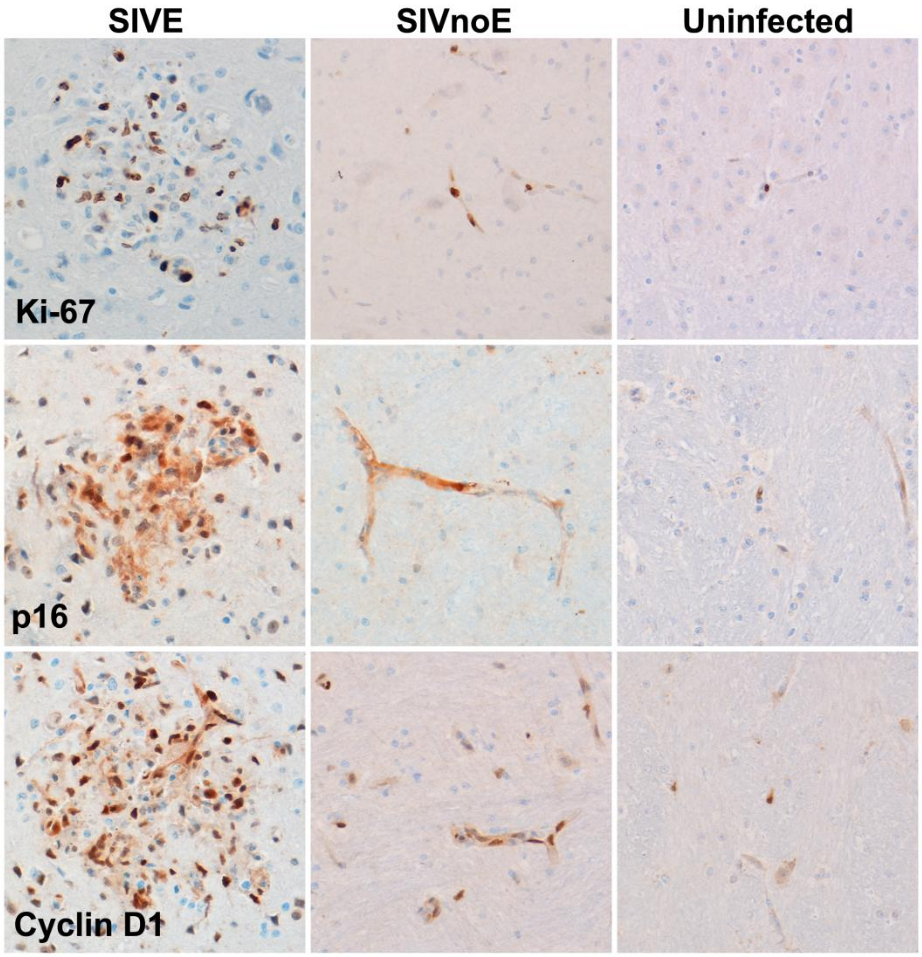

Fig. S1 Proliferation marker expression in the brains of uninfected, SIV-infected, and SIV encephalitic rhesus macaques. Immunohistochemistry for three cell cycle regulators, Ki-67, p16INK4a, and cyclin D1 was performed. Uninfected controls (right column) showed little to no immunoreactivity (DAB, brown) for all three markers, indicating minimal levels of replication. SIVnoE animals (middle column) showed expression in a few scattered cells associated with the vasculature. SIVE animals (left column) demonstrated strong immunoreactivity in the perivascular space and within encephalitic lesions. Sections were counterstained with nuclear stain hematoxylin (blue).

Supplementary Fig. 2

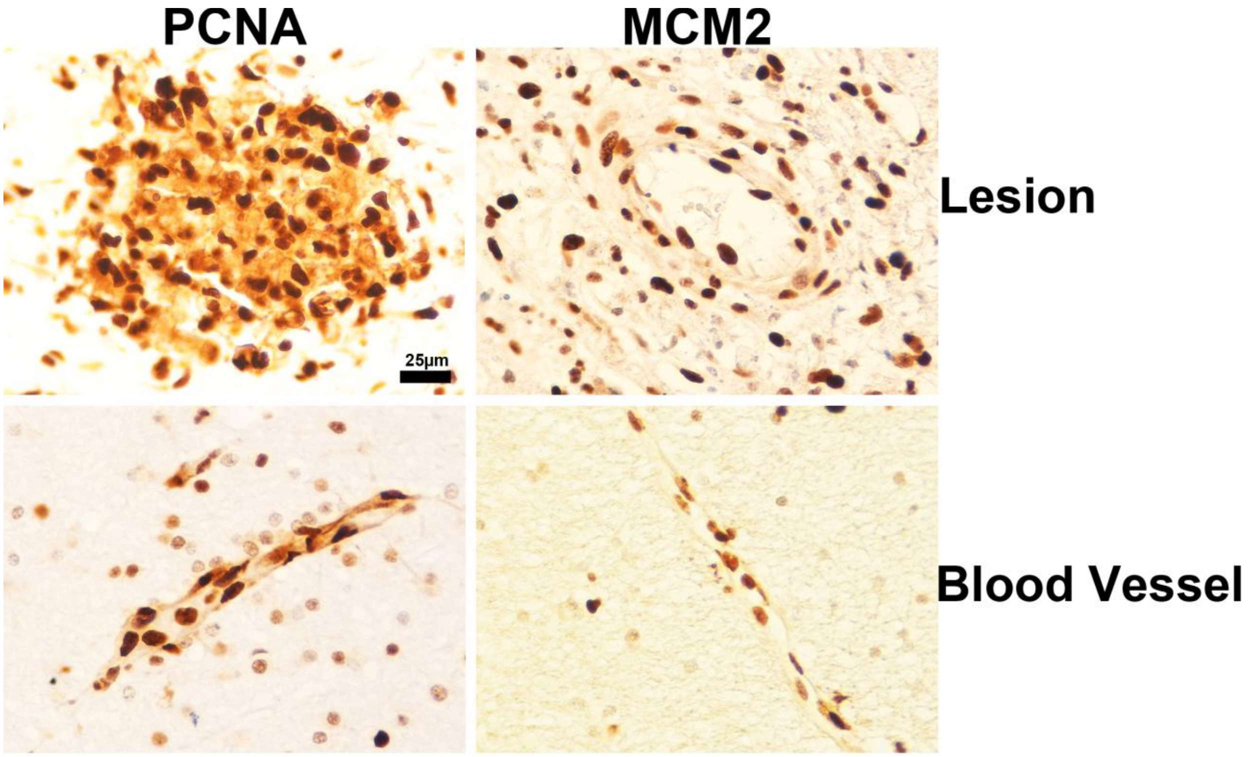

Fig. S2 Immunohistochemical expression of PCNA and MCM2 in SIVE lesions. PCNA (left column) and MCM2 (right column) expression showed positive staining within the encephalitic lesions (upper panel) and around the CNS vessels (lower panel). Magnification, x400.

Supplementary Fig. 3

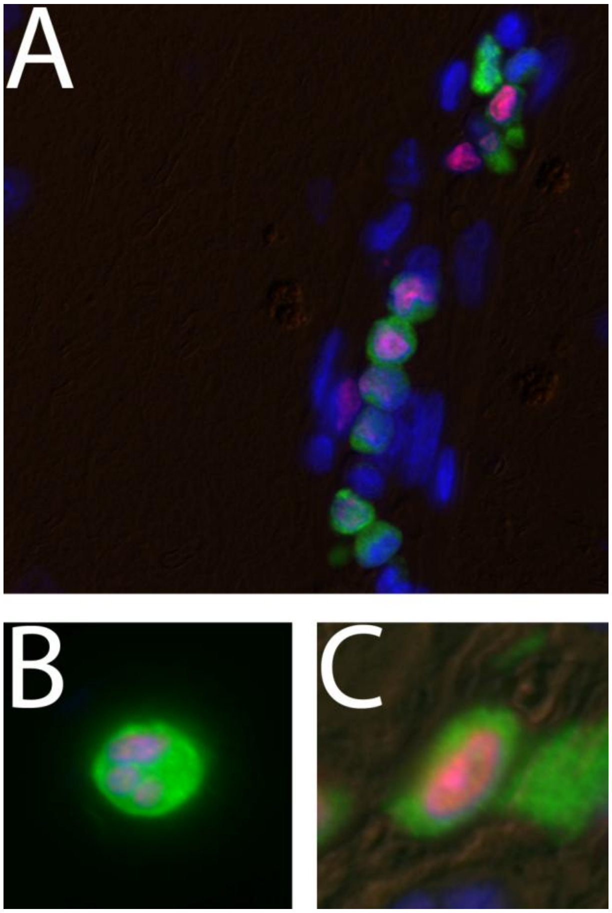

Fig. S3 A minor subset of Ki-67+ cells express MAC387. Triple-label immunofluorescence for Ki-67 (red), MAC387 (green), and DAPI (blue) demonstrated a minor subset of Ki-67+ cells that co-express MAC387 (**a**), a marker that is expressed in polymorphonuclear neutrophils, monocytes and select tissue macrophages. The representative figure presented here illustrates MAC387+Ki-67+ CNS-infiltrating monocytes in addition to MAC387+Ki-67+ polymorphonuclear neutrophils, MAC387+Ki-67− monocytes, and MAC387−Ki-67+ PVM all in and around one vessel. A neutrophil (**b**) and a MAC387+Ki-67+ cell (**c**) are presented in the lower panels.

Supplementary Fig. 4

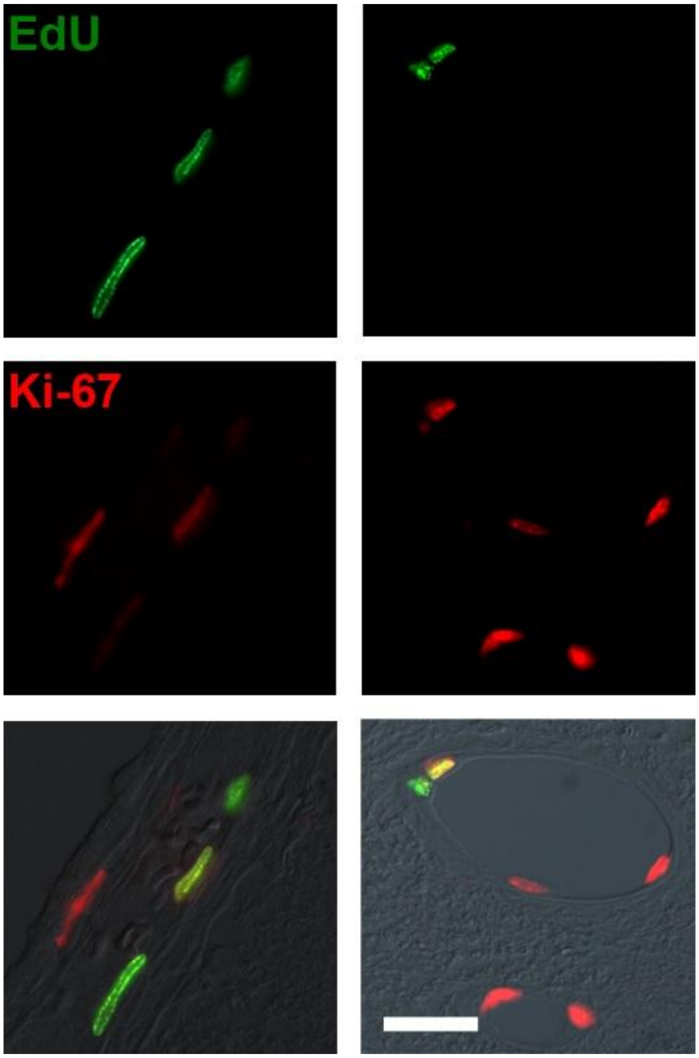

Fig. S4 Incorporation of an additional thymidine analog, EdU, further confirms the proliferative capacity of PVM. Double-label immunofluorescence for Ki-67 (red) and EdU (green) of meningeal (left column) and perivascular (right column) macrophages demonstrated that nearly all EdU-positive nuclei were also Ki-67-positive. Scale bar, 20 μm.

Supplementary Fig. 5

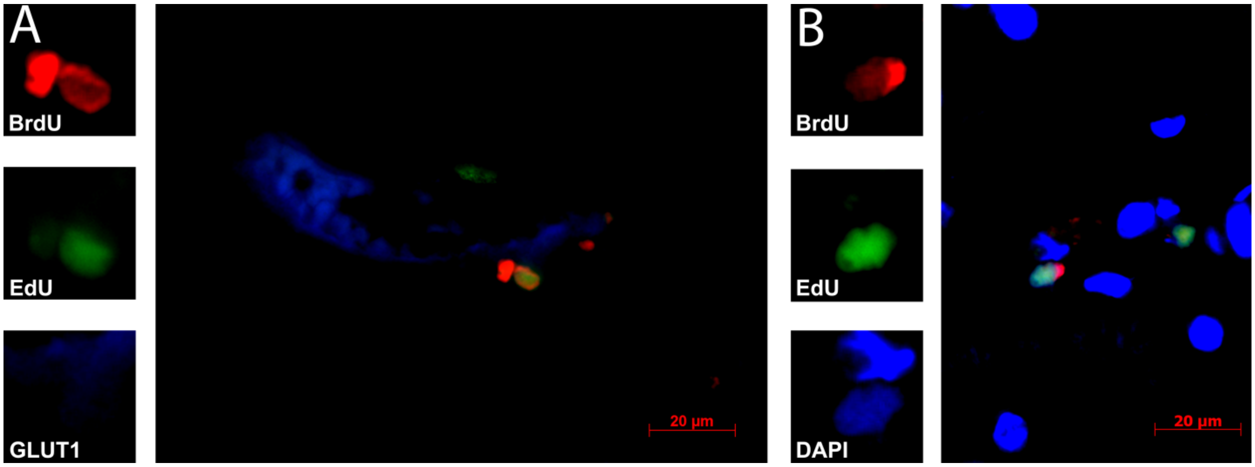

Fig. S5 Triple-label immunofluorescence for BrdU (red), EdU (green), and Glut1 (blue) or DAPI (blue) showed presence of cells with BrdU/EdU double-labeled nuclei around the brain vasculature (**a**) or in the subarachnoid space (**b**) of animals that had received injections of the two analogs at different time points (see Table S1). This was a rare subpopulation, but could point to the ability of some PVM to be long-lasting and capable of self-renewal.

Supplementary Fig. 6

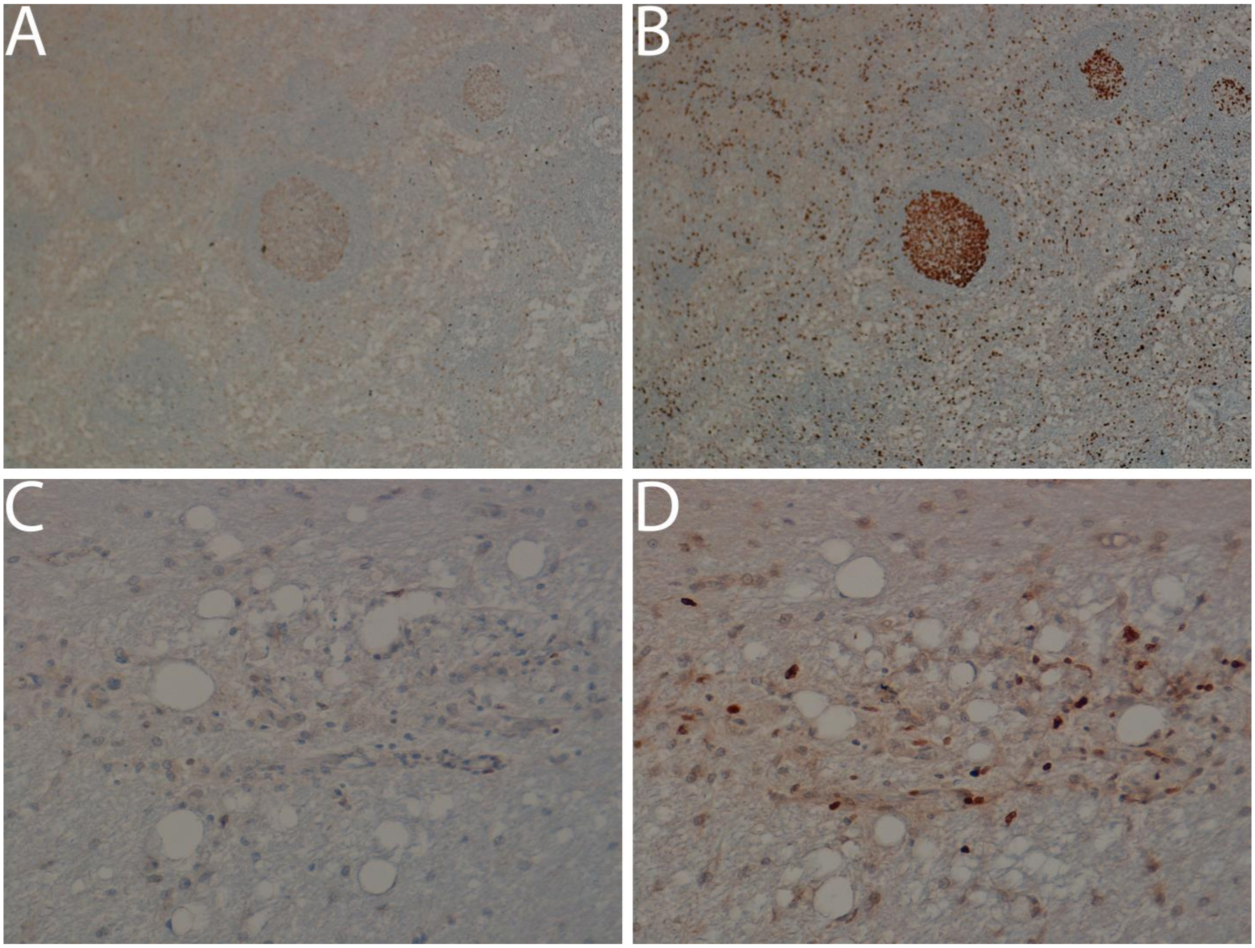

Fig. S6 Comparison of two anti-Ki-67 monoclonal antibodies, clones MIB-1 and SP6, reveals greater immunoreactivity for the latter in macaque tissue sections. Immunohistochemistry for the MIB-1 (**a**, **c**) and SP6 (**b**, **d**) clones of Ki-67 in serial sections of inguinal lymph nodes of normal monkeys (**a**, **b**) and occipital cortex of SIVE monkeys (**c**, **d**) showed weak staining (DAB, brown) for the former and strong reactivity for the latter. In particular, whereas SP6 clearly showed Ki-67+ cells concentrated in the germinal centers of lymph nodes, MIB-1 staining was present but faint. Additionally, Ki-67+ cells and mitotic figures were readily found throughout lesions in SIVE animals when using SP6, while, when using MIB-1, only a few faint Ki-67+ cells could be detected. Sections were counterstained with nuclear stain hematoxylin (blue). Magnifications: x50 (**a**, **b**) and x100 (**c**, **d**). DAB incubation for MIB-1 was 10 min compared to <2 min for SP6.
